# α-Synuclein Oligomers Induced by Docosahexaenoic Acid Affect Membrane Integrity

**DOI:** 10.1371/journal.pone.0082732

**Published:** 2013-11-29

**Authors:** Chiara Fecchio, Giorgia De Franceschi, Annalisa Relini, Elisa Greggio, Mauro Dalla Serra, Luigi Bubacco, Patrizia Polverino de Laureto

**Affiliations:** 1 CRIBI, Biotechnology Centre, Department of Pharmaceutical Sciences, University of Padova, Padova, Italy; 2 Department of Physics, University of Genova, Genova, Italy; 3 Department of Biology, University of Padova, Padova, Italy; 4 Institute of Biophysics, National Research Council of Italy and Bruno Kessler Foundation, Trento, Italy; Cornell University, United States of America

## Abstract

A key feature of Parkinson disease is the aggregation of α-synuclein and its intracellular deposition in fibrillar form. Increasing evidence suggests that the pathogenicity of α-synuclein is correlated with the activity of oligomers formed in the early stages of its aggregation process. Oligomers toxicity seems to be associated with both their ability to bind and affect the integrity of lipid membranes. Previously, we demonstrated that α-synuclein forms oligomeric species in the presence of docosahexaenoic acid and that these species are toxic to cells. Here we studied how interaction of these oligomers with membranes results in cell toxicity, using cellular membrane-mimetic and cell model systems. We found that α-synuclein oligomers are able to interact with large and small unilamellar negatively charged vesicles acquiring an increased amount of α-helical structure, which induces small molecules release. We explored the possibility that oligomers effects on membranes could be due to pore formation, to a detergent-like effect or to fibril growth on the membrane. Our biophysical and cellular findings are consistent with a model where α-synuclein oligomers are embedded into the lipid bilayer causing transient alteration of membrane permeability.

## Introduction

Parkinson disease (PD) is the most common movement disorder, currently affecting approximately 2% of the population older than age 60 years. Hallmarks of PD are the loss of dopaminergic neurons in the substantia nigra (SN) region of the brain and the presence of cytoplasmatic inclusions of α-synuclein (aS) in fibrillar form, known as Lewy bodies and Lewy neurites [1]. aS is a 14 kDa protein that predominantly exists as unfolded monomer under native conditions. Its sequence is characterized by an amphipathic lysine-rich amino terminus, which governs binding to lipids and interactions with membranes [2]; by a hydrophobic central region (NAC, non-amyloid-beta component), responsible for protein aggregation and β-sheet formation [3,4] and a highly acidic C-terminal, rich in Pro and acidic residues. How the fibrillar aggregation of aS is related to PD and neurodegeneration is still an unsolved question. *In vivo* and *in vitro* studies support the hypothesis that oligomers and species formed in the early stage of aggregation of aS, rather than the monomeric or fibrillar protein, represent the toxic species involved in PD [5-7]. Diverse types of oligomers have been described, in terms of structure and toxic activity. It should be mentioned that the methods used for the preparation of oligomers *in vitro* strongly affect their properties, so up to date a robust *in vitro* model of the mechanism by which oligomers exert their toxic activity is not available. 

Although the physiological function of aS is still poorly understood [8], it appears to be involved in modulating synaptic vesicle dynamics [9-11] and may contribute to the activity of controlling synaptic homeostasis-associated proteins [12-14]. Several studies have recently shown a strong link between aS and fatty acids (FAs), in particular brain FAs, such as arachidonic and docosahexaenoic acid (DHA). aS expression affects FAs uptake and metabolism [15,16] and aS interactions with polyunsaturated fatty acids (PUFAs) can rapidly and dynamically affect its oligomerization and further aggregation [17-19]. The appearance of aS toxic oligomers *in vivo* has been linked to the presence of long PUFAs in the brain [20]. Yakunin and colleagues [21] demonstrated that a DHA enriched diet increases the concentration of insoluble aS species in mouse brain. Many evidences suggest a critical role of lipids in both PD and Alzheimer disease, such as changes in activity of phospholipase A_2_ (an enzyme that hydrolyses membrane phospholipids and causes release of FAs) that have been correlated with brain injury, apoptosis and phospholipid metabolism alteration, ultimately leading to neurodegeneration. As a consequence, the interaction between aS and FAs has been proposed to be a key factor in the onset of neurodegeneration and PD [16]. 

In previous studies, we analyzed the aggregation process of aS in the presence of DHA using different protein to DHA molar ratios [19,22]. We demonstrated that DHA exerts an important role in the aggregation of aS, recruiting protein molecules on the droplets surface and being itself part of the aggregates structure. The presence of DHA to the aggregating aS (50:1 mol/mol) leads to the formation of stable oligomers, that are DMSO- and SDS-resistant, do not bind ThT and lack seeding properties, demonstrating that they are off-pathway in the aggregation process of aS. Structurally they contain α-helical and random structure. Furthermore, these oligomers are toxic to cells, compared to aS, suggesting that they are potentially relevant in the pathogenesis of PD.

In the present work we conducted experiments to test the hypothesis that aS oligomers exert their toxic effect by undermining the integrity of lipid membranes. The interaction between aS/DHA oligomers with lipid bilayer was studied by circular dichroism (CD), transmission electron microscopy (TEM) and dynamic light scattering (DLS). We show that aS/DHA oligomers bind to negatively charged membranes and acquire an increased amount of α-helical structure. Upon binding to negatively charged vesicles, oligomers induce leakage of small molecules such as calcein (mw 0.6 kDa) but not of FITC-dextran (mw 10 kDa). Treatment of dopaminergic SH-SY5Y cells with aS/DHA oligomers results in increased permeability to propidium iodide.

## Materials and Methods

DHA and fluorescein isothiocyanate dextran (FITC-Dextran) of average molecular weight of 10,000 Da were purchased from Sigma Chem. Co. (St. Louis, MO). All other chemicals (analytical reagent grade) were obtained from Sigma or Fluka (Buchs, Switzerland). The lipids, 1-palmitoyl-2-oleoyl-sn-glycero-3-phosphocholine (POPC), 1,2-dioleoyl-*sn*-glycero-3-phospho-(1'-rac-glycerol) (DOPG), 1-palmitoyl-2-oleoyl-sn-glycero-3-phospho-L-serine (POPS), 1-palmitoyl-2-oleoyl-*sn*-glycero-3 phosphoethanolamine (POPE) and Brain Total Lipid Extract, were obtained from Avanti Polar Lipids (Alabaster, AL) as chloroform solution and used without further purification.

### aS cloning and expression

The pET28b (Novagen) plasmid was used for the expression of recombinant human aS in *E. coli* BL21 (DE3). The expression and purification of the protein were conducted using the procedure previously described [22]. Protein concentrations were determined by absorption measurements at 280 nm using a double-beam Lambda-20 spectrophotometer (Perkin Elmer, Norwalk, CT). The extinction coefficient of aS at 280 nm is 5960 cm^-1^ M^-1^, as evaluated from its amino acid composition by the method of Gill and von Hippel [23].

### Preparation of oligomers

To prepare oligomeric species, aS was incubated at 37 °C for up to 48 hours at a protein concentration of 50 µM, in PBS (8 mM Na_2_HPO_4_, 137 mM NaCl, 2 mM KH_2_PO_4_, 2.7 mM KCl, pH 7.4) in the presence of DHA (2.5 mM) to obtain a protein/fatty acid molar ratio of 1:50, under shaking at 500 rpm with a thermo-mixer (Compact, Eppendorf, Hamburg, DE). Aliquots of the samples were examined by thioflavin T (ThT) binding assay, CD, TEM, gel filtration (GF) chromatography and RP-HPLC, as previously described [19] to verify the identity and structure of the oligomers and to assure experimental reproducibility. The identity of the eluted material was assessed by mass spectrometry carried out with an electrospray ionization (ESI) mass spectrometer with a Q-Tof analyzer (Micro) (Waters, Manchester, UK). Oligomers were used after purification by GF and concentrated by Amicon Ultra ultrafiltration (10K membrane cutoff) (Millipore, Billerica, MA, USA). 

### Circular dichroism

CD spectra were recorded on a J-710 spectropolarimeter (Jasco, Tokyo, Japan). Far-UV CD spectra were recorded using a 1 mm path-length quartz cell and a protein concentration of 5-7 µM. The mean residue ellipticity [θ] (deg cm^2^ dmol^–1^) was calculated from the equation [θ] = (*θobs*/10) (MRW/*lc*), where *θ*_*obs*_ is the observed ellipticity in deg, MRW is the mean residue molecular weight of the protein, *l* the optical pathlength in cm and *c* the protein concentration in g/mL. The spectra were recorded in PBS buffer, pH 7.4.

### Atomic force and electron transmission microscopy

aS/DHA oligomers were diluted 500 times using Milli-Q water and 10 μl aliquots were deposited on freshly cleaved mica and dried under mild vacuum. Tapping mode AFM images were acquired in air using a Multimode scanning probe microscope equipped with an “E” scanning head (maximum scan size 10 μm) and driven by a Nanoscope IV controller (Digital Instruments, Bruker, Germany). Single beam uncoated silicon cantilevers (type Olympus OMCL-AC160TS, Olympus, Tokyo, Japan) were employed. The tip of the probe was of 7 nm. The drive frequency was between 280 and 300 kHz, and the scan rate was between 0.5 and 1.0 Hz. TEM pictures were taken on a Tecnai G^2^ 12 Twin instrument (FEI Company, Hillsboro, OR, USA), operating at an excitation voltage of 100 kV. Samples for TEM were diluted 2 times and a drop of the solution was placed on a Butvar-coated copper grid (400-square mesh) (TAAB-Laboratories Equipment Ltd, Berks, UK), followed by a drop of uranyl acetate solution (1% w/v).

### Vesicles preparation

To prepare large and small unilamellar vesicles (LUV, SUV), lipids were transferred in glass tubes. Chloroform was dehydrated by gentle helium stream and then warmed at 45°C to remove residual organic solvent. The lipid film was hydrated with PBS pH 7.4 in the absence or presence of calcein (50 mM) or FITC-dextran (1.6 mM) at 40°C for 2 hours with frequent vortexing. Then it was subjected to 5 cycles of freezing and thawing. The suspension of vesicles was extruded 11 times through a 400 nm or 30 nm pore size polycarbonate membrane on lipid extruder (Northern Lipids Inc, Vancouver, BC), in order to obtain LUVs or SUVs. Calcein-loaded and FITC-dextran-loaded vesicles were purified by gel filtration chromatography (Sepharose G-25) to remove unencapsulated dye. The final lipid concentration, determined as total phosphorus, was conducted according to Chen et al., [24]. 

### Dynamic light scattering

The size distribution and the stability of vesicles were checked by DLS experiments, performed on a Zetasizer Nano-ZS instrument (Malvern Instrument, UK). DLS measurements were performed at 25 °C in PBS pH 7.4 in duplicate. During every measurement 12 runs were collected. DOPG LUV and SUV size distributions were measured for vesicles alone and after 30 min of incubation with oligomers. 

### Release assays

For calcein release assay, SUV or LUV were diluted to 50 µM of lipids. After 30 minutes of incubation in the presence of different protein species (monomers, oligomers, fibrils), fluorescence emission at 515 nm was recorded after excitation at 490 nm. Maximum fluorescence emission was obtained by the addition of 0.1% Triton X-100 to disrupt vesicles. The average values of experiments, performed in triplicate, were expressed as a percentage of the maximum effect due to total vesicles disruption. FITC-dextran-loaded SUVs or LUVs were incubated for 30 minutes in the presence of the protein species at a final lipid concentration of 100 µM. Released dye was detected by fluorescence measurement with excitation at 490 nm and emission at 515 nm, after purification of the incubated solution through Microcon (cutoff 50000) to allow FITC-Dextran elution. The minimum and maximum effect was obtained by measurement of a liposome solution respectively with or without 0.1% Triton X-100. The average values of experiments, performed in triplicate, were expressed as a percentage of the maximum effect calculated as total vesicles disruption obtained with Triton X-100.

### Cell permeabilization assay

To examine whether aS/DHA oligomers can permeabilize cell membranes, we exploited the ability of propidium iodide (PI; MW 668.4 Da) to bind DNA as indirect reporter of membrane damage. Dopaminergic SH-SY5Y cells were cultured in 24-well plates (seeding density ~50000 cells/well) and twenty-four hours after seeding treated with 0.5 µM of aS monomer or aS/DHA oligomers. Impermeant dye PI (2 µg/ml) and counterstain dye Hoechst 33242 (2 µg/ml) were added to cells simultaneously to the treatment. After a 30 min-incubation at 37°C, PI-positive cells were counted in three replicate cultures, acquiring an average of 5 fields per culture (250-350 cells/field). The experiment has been repeated 3 times independently. Saponin (50 µg/ml) was used as positive control of membrane permeabilization.

### Aggregation study

In order to induce aggregation, a 50 µM solution of aS/DHA oligomers (calculated as monomer concentration) was incubated in 20 mM Tris∙HCl, 150 mM NaCl pH 7.4 in the absence or in the presence of DHA (2.5 mM, molar ratio 1:50) or DOPG SUVs (1 mM or 2.5 mM, molar ratio protein/lipid 1:20 and 1:50) at 37°C, under shaking at 500 rpm with a thermo-mixer (Compact, Eppendorf, Hamburg, DE). Aliquots of the samples were examined by ThT binding assay, CD and TEM. The ThT binding assays were performed accordingly to LeVine [25] using a 25 μM ThT solution in 25 mM sodium phosphate (pH 6.0). Aliquots (30 μl) of protein samples containing aggregates were taken at specified times and diluted into the ThT buffer. Fluorescence emission measurements were conducted at 25°C using an excitation wavelength of 440 nm and recording the ThT fluorescence emission at 484 nm. 

### Planar Lipid Membrane experiments

Solvent-free Planar Lipid Membrane (PLM) was composed of equimolar mixture of 1,2-dioleyl-phosphatidyl-glycerol (DOPG) and 1,2-dioleyl-phosphatidyl-ethanolamine (DOPE) and formed on an aperture in a 25 μm thick Teflon septum separating two chambers, as described in Dalla Serra et al. [26]. Ionic currents were recorded by a patch clamp amplifier (VA-10X npi, Tamm, Germany), filtered at 100 Hz, digitalized and acquired at 2 kHz by the computer using DigiData 1322 A/D converter and pClamp software (Axon Instruments, Sunnyvale, CA). 

## Results

### Biophysical properties of oligomers

**Figure 1:**
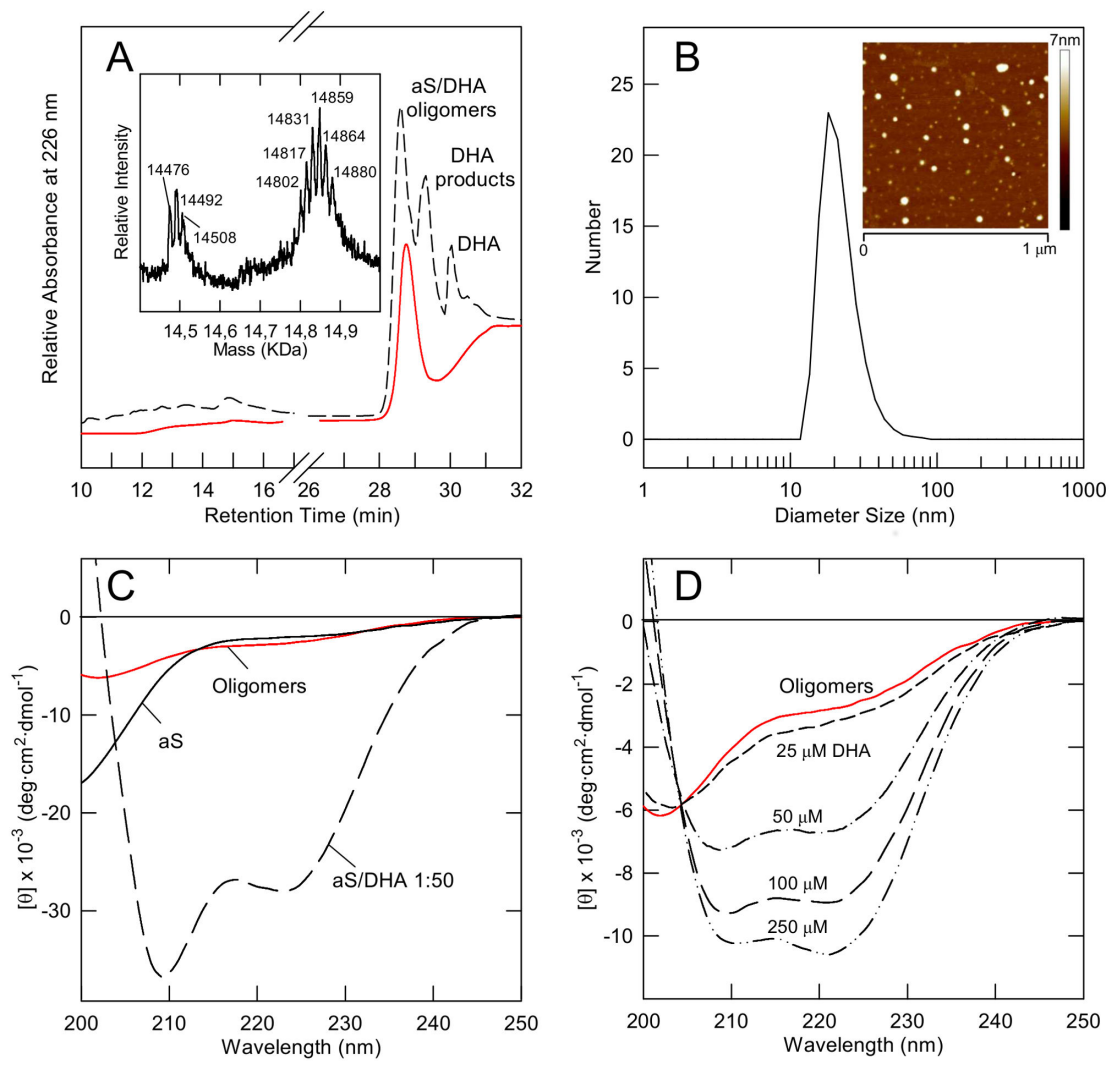
Chemico-physical characterization of aS/DHA oligomers after their isolation by gel filtration. (A) RP-HPLC of aS/DHA oligomers after their isolation by gel filtration (red line) and before (dashed line). HPLC analyses were conducted using a Jupiter C4 column (4.6 x 150 mm; Phenomenex, CA, USA), eluted with a gradient of acetonitrile/0.085% TFA vs water/0.1% TFA from 5% to 38% in 5 min, from 38% to 43% in 15 min, recording the absorbance at 226 nm. The identity of the eluted material was assessed by mass spectrometry and the spectrum of oligomers is reported (inset). (B) DLS for particle size estimation of aS/DHA oligomers. (Inset) Tapping mode AFM image of oligomers. Scan size was 1.0 µm; Z range was 7 nm. (C) Far UV CD of oligomers after purification by gel filtration (red line). The spectra of monomeric aS and aS in the presence of DHA (protein/DHA 1:50) are also reported as reference. (D) Titration experiment by far UV CD of oligomers in the presence of increasing amount of DHA. The numbers close to the spectra indicate the amount (µM) of DHA.

aS oligomers, induced by DHA, were obtained as previously described [19,22] and were purified by gel filtration from monomeric aS, after 48 h incubation [19]. The fraction corresponding to oligomers was analyzed by RP-HPLC (Figure 1A, red line) and compared with RP-HPLC profile of the same sample before its purification by gel filtration. The peak (RT 29.6 min) corresponding to DHA is lacking, indicating that exchangeable DHA is removed. Mass spectrometry (Figure 1A, inset) shows that aS molecules in the oligomers are chemically modified for the presence of covalently bound DHA, as previously determined [19]. Previously, TEM analysis showed that the chromatographic fraction corresponding to oligomers had a spherical morphology, with diameters ranging from 12 to 35 nm [19]. In Figure 1B the number size distribution from aS/DHA oligomers obtained by DLS analysis is shown, indicating a mean size of 22±8 nm. AFM measurement (inset), based on height differences, shows the presence of smaller (1.1±0.1 nm) and larger (4.1±0.1 nm) oligomers. 

Spectroscopic analysis by far UV CD of oligomers after purification by gel filtration is reported (Figure 1C). The spectrum of oligomers (red line) is shown in comparison with CD spectra of monomeric aS (continuous line) and of the mixture of aS/DHA before gel filtration (dashed line, see ref. 19). This analysis indicates that oligomers acquire a partly folded conformational state with a moderate content of α-helical structure. Further addition of DHA to oligomers, isolated by gel filtration, induces an increase of their α-helical structure (Figure 1D), as observed for monomeric aS [22]. The transition between partly folded state and α-helix follows a two-state model, for the presence of an isodichroic point at ~203 nm in the titration experiment. 

### Oligomers interact with negatively charged membranes

**Figure 2:**
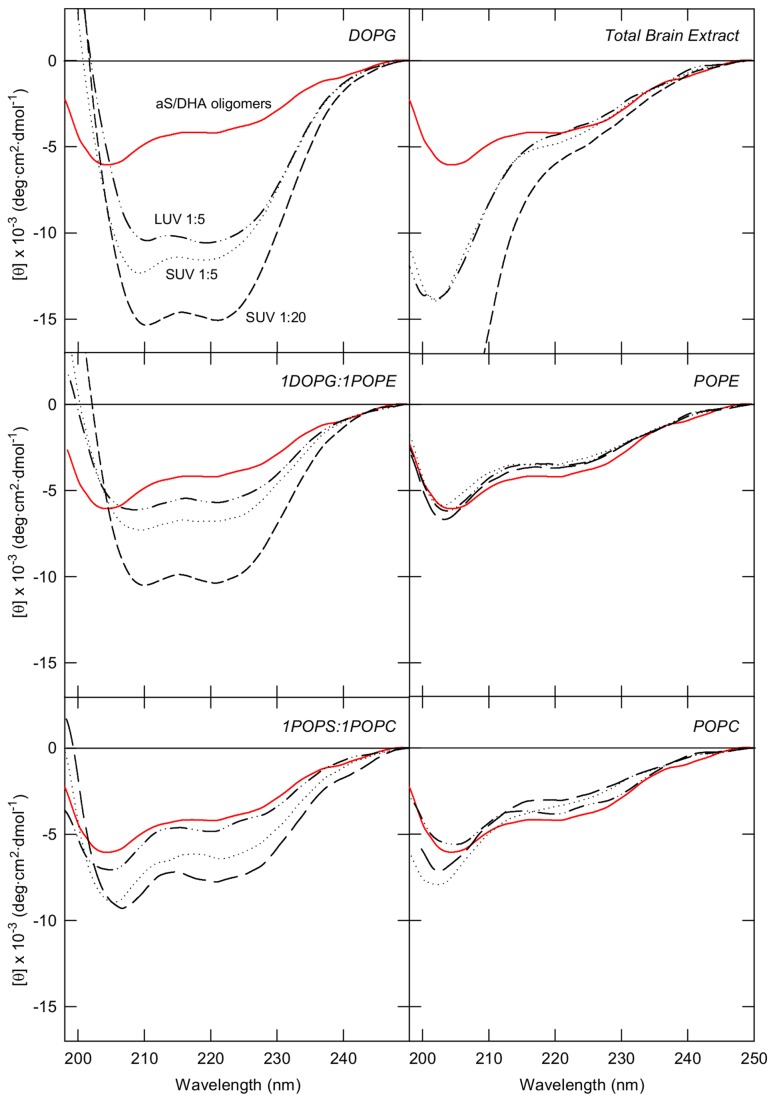
Interaction of aS/DHA oligomers with lipid vesicles with different composition monitored by far UV CD. The spectra relative to aS/DHA oligomers alone (red continuous line), aS/DHA oligomers in the presence of SUV at molar ratio 1:5 (dotted line) and 1:20 (dashed line) and in the presence of LUV at molar ratio 1:5 (dotted-dashed line) are reported.

In order to elucidate the mechanism by which DHA/aS oligomers exert their toxic activity, the interaction with model membranes has been studied. CD spectroscopy was used to determine the effects of lipid binding on the secondary structure of aS/DHA oligomers and various combinations of charged and uncharged lipids have been used. In Figure 2 the CD spectra of aS/DHA oligomers recorded in the presence of SUV or LUV are reported. aS/DHA oligomers interact with synthetic membranes containing negatively charged DOPG, or a mixture of negative and neutral phospholipids as 1DOPG:1POPE and 1POPS:1POPC (Figure 2), as more pronounced minima at 208 and 222 nm are observed in the spectra upon binding to these membranes, indicating an increase of α-helical conformation. SUV or LUV containing neutral phospholipids (POPC or POPE) do not induce any conformational change in aS oligomers, as well as vesicles constituted by a mixture of lipid extracted from brain (Figure 2). SUV and LUV with the same composition exert similar effect on oligomers secondary structure suggesting that curvature is not a discriminating factor.

### Oligomers do not aggregate in the presence of lipids and membranes

**Figure 3:**
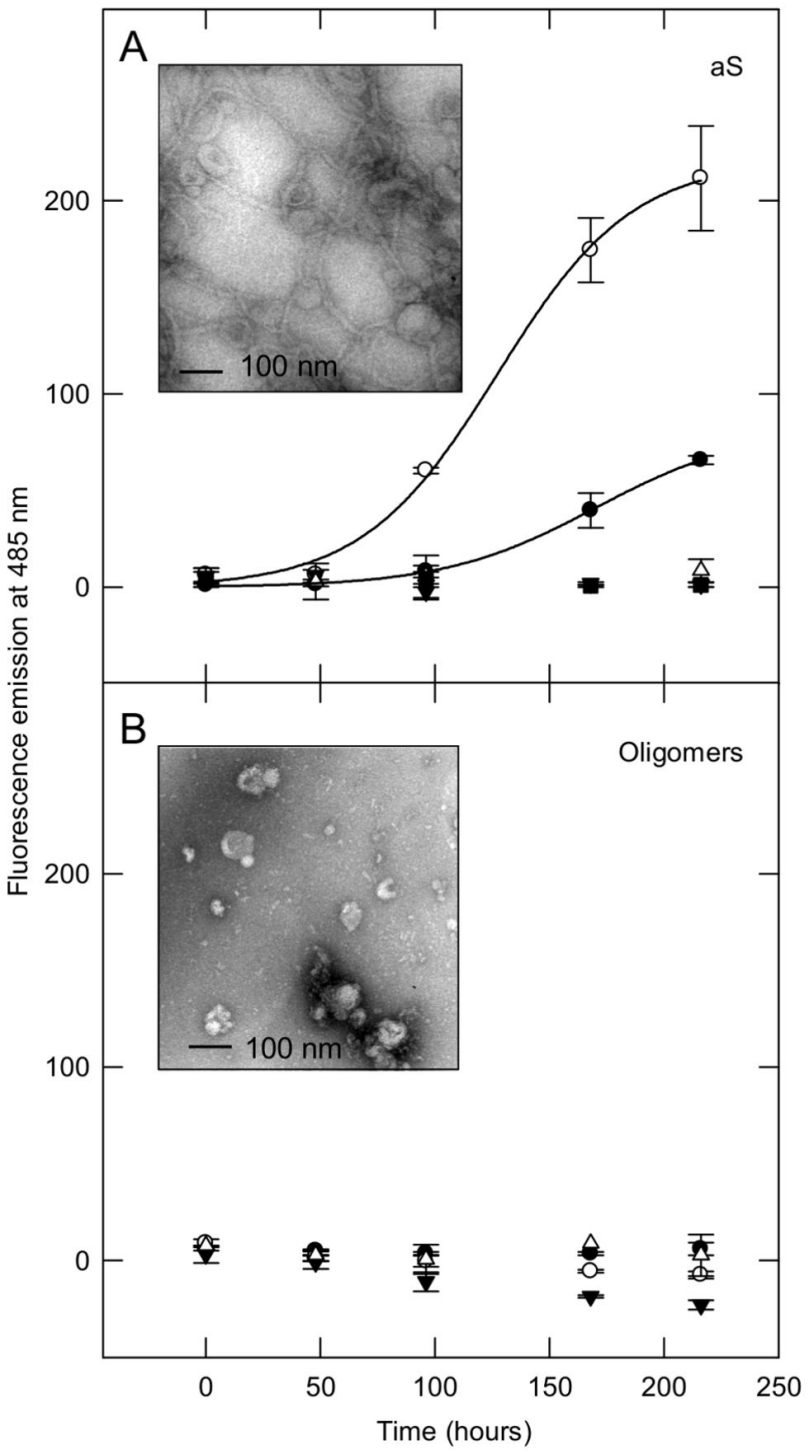
Aggregation studies. aS (A) and aS/DHA oligomers (B) aggregation process at 37°C in the presence of lipids, followed by ThT binding assay. aS or aS oligomers were dissolved in 20 mM Tris, 150 mM NaCl pH 7.4 at a 50 µM concentration in order to induce aggregation, in the absence (black circles) and in the presence of DOPG SUV, at molar ratio 1:20 (empty circles), 1:50 (empty triangles) and in the presence of DHA (molar ratio 1:50, inverted black triangles). The excitation wavelength was fixed at 440 nm, and the fluorescence emission was collected at 485 nm. To better visualize the aggregation trend of aS and aS in the presence of DOPG (molar ratio 1:20), the data points are fitted with a sigmoidal equation (SigmaPlot software). Inset: TEM images of protein material relative to aS and aS/DHA oligomers samples after 9 days of incubation in the presence of DOPG SUV (molar ratio 1:20).

Membrane-induced or lipid-accelerated fibril formation of amyloidogenic proteins was frequently observed [27-29]. To analyze the effect of membranes on the aggregation properties of aS/DHA oligomers, aS and aS oligomers were incubated in the absence and in the presence of vesicles (molar ratio protein/lipid of 1:20 and 1:50). The aggregation was conducted in the presence of DOPG SUV, since oligomers preferentially interact with negatively charged membranes and DHA (molar ratio protein/fatty acid 1:50), as control. The effect of the lipids on protein aggregation was monitored by ThT fluorescence assay (Figure 3). The aggregation process of aS results sped up in the presence of DOPG SUV (1:20) (Figure 3A, empty circles), while in the presence of DOPG SUV (1:50) is inhibited (Figure 3A, empty triangles). TEM analysis show that in the mixture corresponding to aS incubation for 9 days in the presence of SUV DOPG (1:20) there are individual aS amyloid-like fibrils associated to the surface of SUV (Figure 3A, inset). The size and morphology of these fibrils are similar to those of aS fibrils formed in the absence of SUV. In both cases, aS forms straight, unbranched fibrils with a width of 10–15 nm. An additional feature is that the shape of vesicles in contact with fibrils is not changed. In the presence of DHA, as previously observed, there is not formation of aS aggregates that bind ThT dye (Figure 3A, inverted black triangles). In the case of aS/DHA oligomers, their incubation in the presence or the absence of SUV or DHA does not result in any increase of the fluorescence of ThT dye, confirming their nature of off-pathway intermediates (Figure 3B). The aggregation process was further monitored by CD spectroscopy, confirming the presence of β-sheet structure only for ThT-positive fibrils (data not shown). 

### Oligomers induce permeabilization of artificial membranes

**Figure 4:**
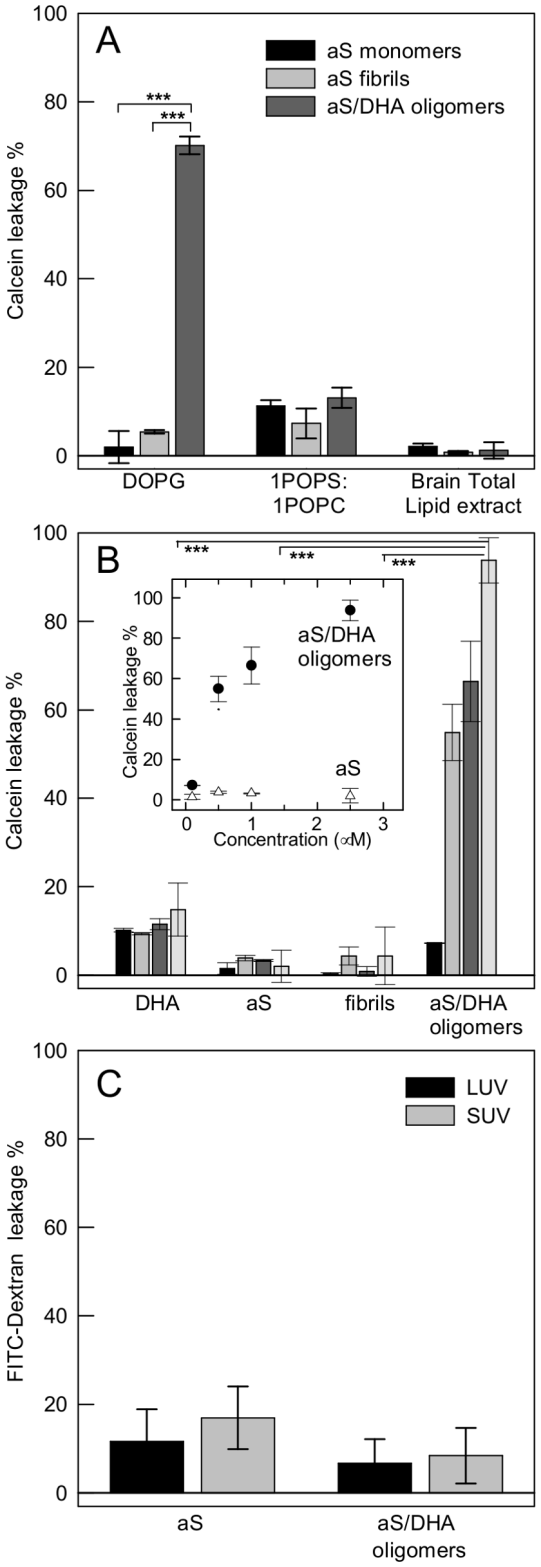
Leakage assays. (A) Calcein efflux from 50 µM LUV of different lipid composition induced after 30 min by monomeric, fibrillar and oligomeric aS at a protein/lipid molar ratio of 1:20. (B) Calcein efflux from 50 µM DOPG SUV upon addition of increasing amount (0.1, black bars; 0.5, grey bars; 1, dark grey bars; 2.5 µM, light grey bars) of protein species. Inset: Dependence of the leakage from 50 µM DOPG SUV on the concentration of protein or oligomers. The effect induced by increasing concentration of monomeric aS is also reported. (C) FITC-dextran leakage from 50 µM DOPG LUV and SUV induced after 30 min of incubation by aS/DHA oligomers and aS. Leakage is expressed as percentage of the maximum possible effect induced by the addition of Triton X-100. Statistical significance was calculated by student’s t-test (*p*<0.001).

The calcein leakage test was performed to detect changes in membrane permeabilization as result of oligomers binding. The amount of calcein release from LUV or SUV was determined after 30 min of incubation with the oligomers. In Figure 4A the leakage of calcein from LUV was shown in comparison with the effect induced by monomeric and fibrillar aS. We used LUV containing phospholipids which interact (DOPG), partially interact (1POPC:1POPS) or do not interact (TBE) with oligomers, as shown by CD measurements. It is evident that oligomers are able to induce a significant dye release, in comparison to monomeric aS and fibrils. The effect is selective for negatively charged membranes. This effect was also analyzed on DOPG SUV using different protein/lipid molar ratio (Figure 4B). The leakage occurs in a dose-dependent manner and reaches about 50% at 0.5 μM protein concentration (Figure 4B, inset). The permeabilizing activity of aS/DHA oligomers shows selectivity as a function of molecular dimension of the dye, indeed calcein passes through the membrane in the presence of oligomers, while larger molecules such as FITC-dextran with a Stoke’s radius 1.9-2.3 nm do not (Figure 4C). By the way, this value roughly defines an upper limit to the pore radius.

### Oligomers increase permeability of dopaminergic cells

**Figure 5:**
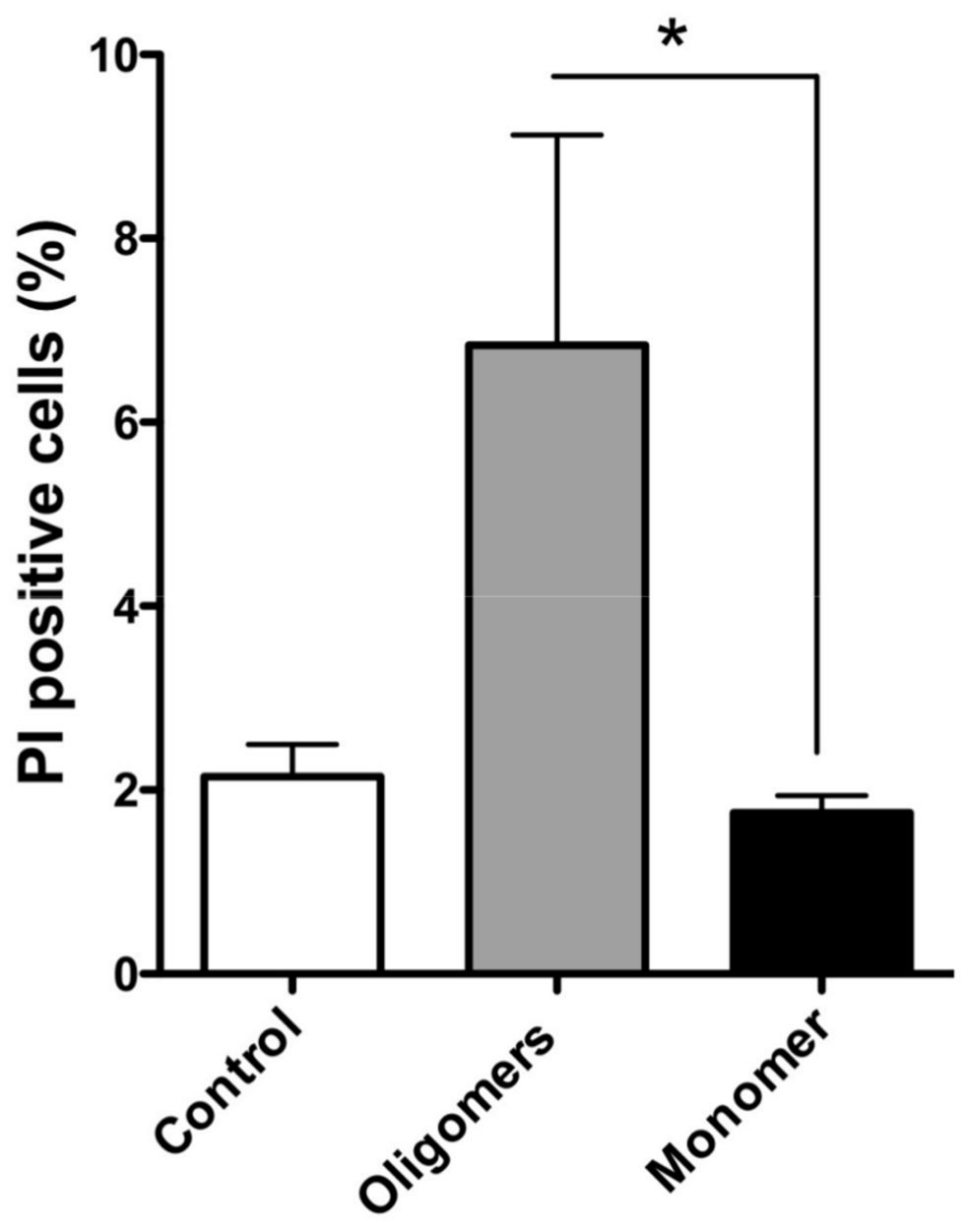
PI influx in dopaminergic cells. SH-SY5Y cells were treated with aS/DHA oligomers and the number of PI-positive cells was calculated as a percentage of the total counterstained cells. Quantitation (n = 3 cultures, with 5 fields analyzed per culture, 250-300 cells per field; error bars indicated the S.E.) indicated that aS/DHA oligomers increased the percentage of PI-positive cells. Statistical significance was calculated by one-way ANOVA (p < 0.05) compared with mock control or monomeric aS.

Although we clearly see that aS/DHA oligomers are capable of permeabilizing artificial membranes, we next asked whether this activity is also observed in a more complex system such as the cell membrane. We used PI, a cell-impermeant DNA dye, to evaluate the effect of oligomers to modify membrane permeability of dopaminergic SH-SY5Y cells. SH-SY5Y cells treated with both aS oligomeric aggregates purified by gel filtration and monomeric aS were observed within 1 hour. We observed a modest but significant increase of PI-positive cells upon treatment with aS/DHA oligomers compared to monomer or vehicle control as quantified by one way ANOVA with Tukey’s post hoc test (*, P<0.05; Figure 5).

### Oligomers do not exert detergent-like effect

**Figure 6:**
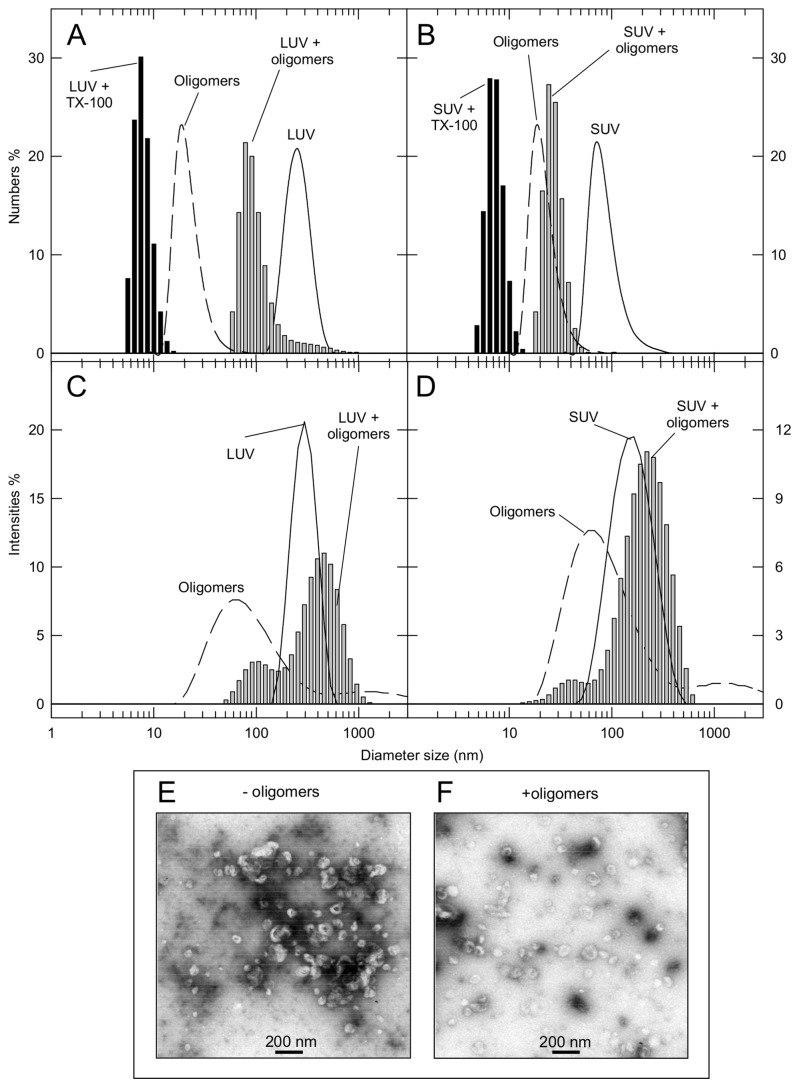
Interaction of aS/DHA oligomers with DOPG vesicles by DLS and TEM. DLS analysis, expressed as numbers (A,B) or as intensities (C,D) and TEM images (E,F) are reported. DLS measurements of DOPG LUV (A,C) and SUV (B,D) are conducted in the absence of oligomers (continuous lines), and upon 30 min-addition of oligomers (molar ratio 1:20, light grey bars). aS/oligomers alone are represented by dashed lines. The effect induced by Triton X-100 on LUV and SUV is reported (A,B, dark grey bars). TEM images of DOPG SUV (E) or in the presence (F) of aS/DHA oligomers were taken after 30 min of incubation.

To test the possibility that aS/DHA oligomers were able to compromise the integrity of the membrane by a detergent-like mechanism, large and small unilamellar vesicles were analyzed by dynamic light scattering (DLS) after incubation with oligomers for 30 minutes, using a molar ratio protein:lipid of 1:20. In Figure 6 the numbers and intensities size distributions of DOPG LUV (A,C) and SUV (B,D) before and after addition of aS oligomers are shown. The peak relative to vesicles alone (solid lines) is centered at 270±10 nm for LUV (A) and at 83±8 nm for SUV (B). The mean size of oligomers alone (dashed line) is 22±8 nm. The addition of oligomers to vesicles induces a change in the size distribution of vesicles (grey bars) different from that induced by Triton X-100 (A-B, black bars). Since the addition of Triton results in disaggregation of the vesicles into micelles, we can conclude that the oligomers do not act as detergents. To have a better view of the size distribution of vesicle upon addition of oligomers, the measurements expressed as intensities are also reported (C,D). The peak relative to vesicles splits into two peaks (gray bars) corresponding to two vesicles population with increased (centered at ~470 nm for LUV and at ~235 nm for SUV) and decreased size (centered at ~135 nm for LUV and at ~35 nm for SUV). This resizing can be ascribable to clustering/fusion of the vesicles in the case of the increased size or to remodeling in the case of the smaller species. Of interest, the same experiment was followed by TEM (Figure 6 E,F), that does not evidence substantial difference in vesicles shape after oligomer addition.

### Oligomers activity on planar lipid membrane

**Figure 7:**
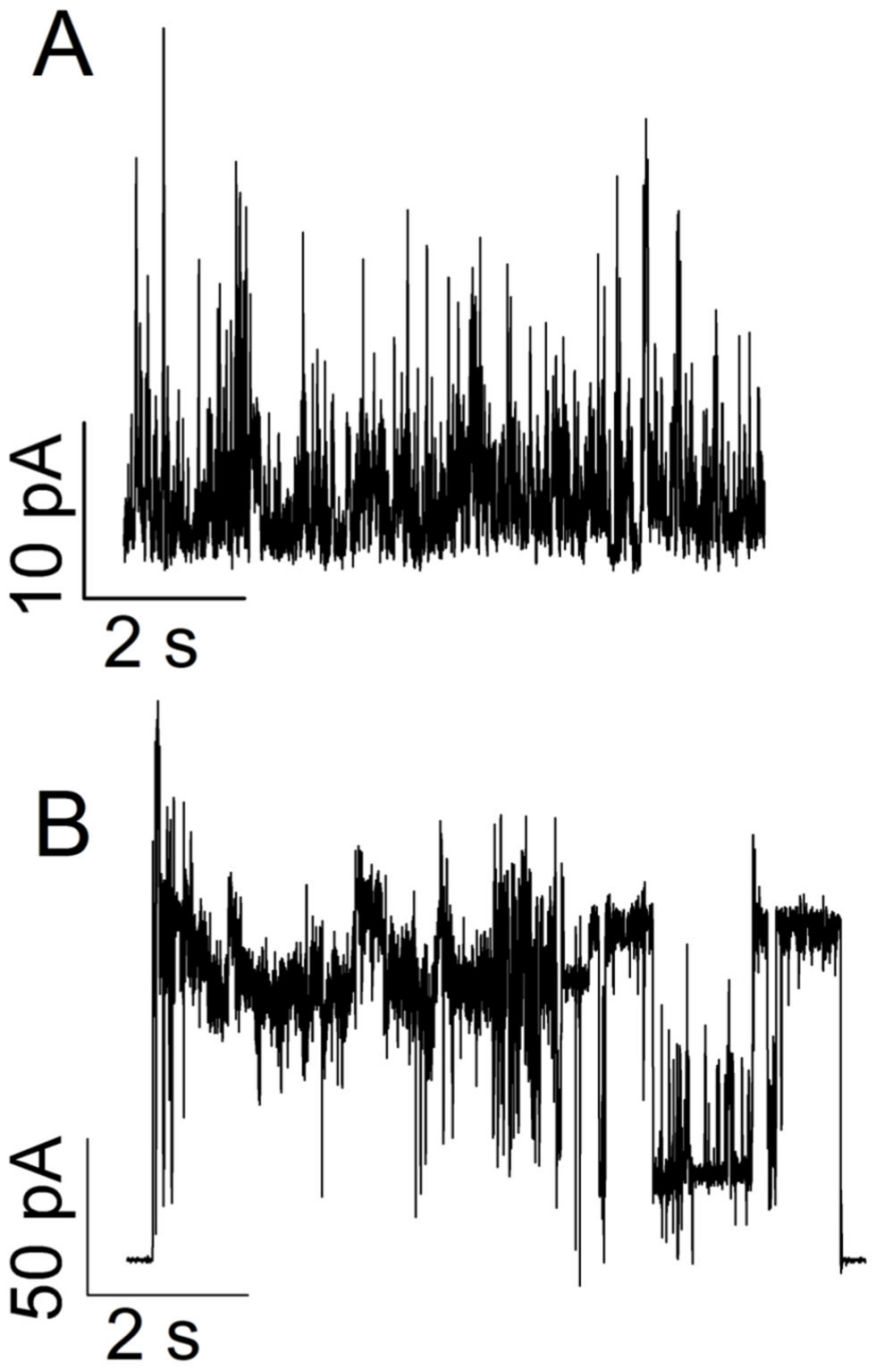
PLM experiments. Electrophysiological activity of aS/DHA oligomers on DOPG/DOPE:1/1 lipid membranes. Buffer composition was KCl 100 mM, Hepes 5 mM, pH 7.0. Protein concentration was 68 nM. Applied potential +80 mV (A) or +100 mV (B).

aS/DHA oligomers at a final concentration of 68 nM are able to increase the conductance of PLM composed of DOPG/DOPE:1/1 upon the application of either positive or negative high potentials (absolute values larger than 100 mV). In 50% of experiments current fluctuations appeared non structured with short living spikes with small ionic currents (roughly 10 pA at +80 mV) as reported in Figure 7A. This permeabilizing activity generally disappeared after few minutes without causing membrane rupture, with protein concentration up to 380 nM. In 25% of experiments a more intense current, up to 100 pA, has been recorded as reported in Figure 7B. Sometimes more stable apertures can also be seen as reported in the right side of Figure 7B. In this case the current-voltage curve has a non-ohmic characteristic with higher current at negative applied voltages. No membrane destabilization has been recorded in the remaining 25% of experiments. Control experiments with DHA alone (up to 38 μM in EtOH) did not show any membrane perturbation (not shown).

## Discussion

There is growing interest in understanding a possible link between aS toxicity in synucleinopathies and aS-lipid interaction [16,29,30]. aS toxicity is indeed associated with alteration in vesicle trafficking [31], mitochondrial function [32], and lipids, especially brain FAs biosynthesis and metabolism [33]. Increasing evidence shows that oligomers originating in early stages of aS fibrillation are responsible for toxicity. It has been shown that elevated brain DHA levels accelerate aS accumulation and oligomerization [20] leading to neurotoxicity [21]. We produced *in vitro* oligomeric species of aS in the presence of DHA, which resulted toxic in cultured dopaminergic cells [19]. Here we have studied their activity on membranes in an attempt to understand their mechanism of toxicity. The latter is object of debate in the literature for the difficulty to define a unifying picture of the toxic activity. The controversy springs from both the transient nature of aS oligomers and from the many different types of oligomers that have been described, a consequence of the variety of the method of preparation. In the presence of DHA, it is induced the formation of relatively stable oligomers that are off-pathway in aS aggregation process. 

aS oligomers have been described as annular and spherical-shaped and are generally characterized by beta-sheet structure, some of them resulted toxic to cells [6,34-36]. Based on TEM and AFM measurements, aS/DHA oligomers share similar size with diameters ranging from 12-30 nm. A specific difference with oligomers described above resides in the secondary structure. aS in the presence of DHA acquires α-helical structure [22] and oligomers, obtained upon aS/DHA incubation (3-48 hours), maintain α-helical structure. After a gel filtration purification step, exchangeable DHA, not the covalently bound one, is removed and with it the effect on oligomers structure. Consequently, part of the DHA dependent secondary structure is lost, and the oligomers acquire a partly folded state, significantly different from the natively unfolded structure of monomeric aS. These partly folded oligomers are able to acquire again α-helical structure upon addition of DHA or in the presence of SUV or LUV containing negatively charged head groups. A similar behavior was previously observed for aS [2,27,37-40]. In monomeric aS the N-terminal region is essential for membrane recognition and for cooperative formation of helical domains [41]. So it is reasonable to assume that in the partly folded state, the N-terminal region or part of it is free to drive the association of oligomers with membranes and lipids. On the other hand, the NAC region (residues 61-95) seems not available or hidden in the interior of the oligomer in view of the fact that these species do not aggregate even in the presence of membranes. Indeed the membranes can affect the fibril formation process of aS and other amyloidogenic proteins [27,28]. Fibrillation can be accelerated or even modulated by lipids and membranes and this strictly depends on the relative protein/lipid ratio [42-44]. In conclusion aS/DHA oligomers have two main structural features: the conformational sensitivity to environment for the ability to interact with lipids and to undergo structural transition, and secondly the stability under conditions that favor aggregation. The overall structure of the oligomers could be stabilized by hydrophobic interactions deriving from the association of NAC regions. 

An interesting property of aS/DHA oligomers is their ability to permeabilize membranes, as verified using a leakage assay from unilamellar phospholipid vesicles and ionic current measurements. Also dopaminergic cells membranes are perturbed as treatment with oligomers significantly increases the number of cells internalizing the membrane-impermeant dye propidium iodide. These results suggest that membranes can be a possible target of aS oligomers activity. This can be particularly harmful for neuronal cells, where electrical activity and action potential firing are finely regulated by transient, voltage-gated channel-mediated variation of membrane potential and intracellular calcium concentration. Indeed, increased membrane permeability could result in a variety of intracellular processes and leads to excitotoxicity, as well as dissipation of sodium and potassium gradients with deleterious impact on electrical neuronal activity. Several mechanisms have been proposed to explain oligomers activity on membranes, such as the pore-like mechanism [34], a thinning effect due to lipid extraction from the bilayer [28], a complete disruption of the membrane [45] or a transient membrane destabilization [46]. The pore-like hypothesis is especially accepted for those proteins forming oligomers or protofibrils with annular or ring-like structure [44,47]. In our case the leakage activity tested on synthetic liposomes shows selectivity of markers size, evidencing that the average dimension of the aperture should be between 1 and 4 nm [48]. The permeabilization effect is induced in cells just after 30 min of treatment. These observations would be consistent with the pore-like mechanism [44]. Nevertheless the activity of oligomers on a planar lipid membrane system seems due to a non-specific membrane permeabilization rather than to the formation of structured membrane apertures like those previously demonstrated for monomeric aS [49]. DLS and microscopy measurements allow also to exclude a detergent-like activity of aS/DHA oligomers on phospholipids vesicles. Indeed DLS measurements of both LUVs and SUVs in the presence of aS/DHA oligomers show that the vesicles size distribution is modified, even if without changes to the overall vesicles morphology. 

We are analyzing a population of oligomers that contain a source of heterogeneity deriving from the presence of covalently bound DHA and additionally of oxidative modifications [19]. The degree of chemical modification of aS molecules embedded in the oligomers is very likely to affect the affinity for and the permeabilizing activity on membranes. We can reasonably hypothesize that these modifications occur especially at the level of His or Lys residues localized in the N-terminal region of aS [50,51]. Therefore the charge distribution of the basic KXKE repeats of the N-terminal region of aS/DHA oligomers could be altered, affecting the interaction with membrane that is primarily mediated by a charge effect [2,46].

In conclusion, aS/DHA oligomers interacting with negatively charged membranes induce a perturbation of the phospholipid bilayer with the release of encapsulated small molecules. Oligomer molecules remain entrapped on the vesicles, as peripheral membrane proteins, acquiring α-helical structure. Damaging of membrane structural integrity seems to be an essential step in the cytotoxic activity of aS oligomers formed in the presence of DHA.
